# Seasonal variation in molecular and physiological stress markers in Asian elephants

**DOI:** 10.1093/conphys/coad029

**Published:** 2023-05-19

**Authors:** Susanna Ukonaho, Vérane Berger, Diogo J Franco dos Santos, Win Htut, Htoo Htoo Aung, U Kyaw Nyeing, Sophie Reichert, Virpi Lummaa

**Affiliations:** Department of Biology, University of Turku, Vesilinnantie, 5, Turku 20014, Finland; Department of Biology, University of Turku, Vesilinnantie, 5, Turku 20014, Finland; Department of Biology, University of Turku, Vesilinnantie, 5, Turku 20014, Finland; Myanma Timber Enterprise, MONREC, Myanmar; Myanma Timber Enterprise, MONREC, Myanmar; Myanma Timber Enterprise, MONREC, Myanmar; Department of Biology, University of Turku, Vesilinnantie, 5, Turku 20014, Finland; Department of Biology, University of Turku, Vesilinnantie, 5, Turku 20014, Finland

**Keywords:** seasonality, oxidative stress, lymphocytes, heterophils, faecal cortisol metabolites, body condition, Asian elephants

## Abstract

Free-living species exhibit seasonal variation in various life history traits, including vital rates such as birth and death patterns. Different physiological mechanisms are thought to underlie the expression of life history traits that contribute to lifetime fitness. However, although the broad impacts of seasonality on life history traits and trade-offs is well established in many systems, the exact physiological mechanisms responsible for driving differences within and between individuals are poorly understood. Among them, molecular and physiological stress pathways, such as stress hypothalamic–pituitary–adrenal axis and oxidative stress, have potential to mediate relationships between individual survival, reproduction and environmental seasonality. Here, we determine how different physiological markers of stress including faecal cortisol metabolites (FCMs), heterophils/lymphocytes (H/L) ratio, two markers indicating oxidative balance including a marker of oxidative damage (reactive oxygen metabolites, ROM) and a marker of antioxidant defences (superoxide dismutase, SOD) and body weight vary in a large semi-captive population of Asian elephants (*Elephas maximus*) exposed to extreme seasonality (e.g. elevated temperatures). Individuals showed higher FCM levels and H/L ratios during cold season, indicating increased stress, and the lowest FCM levels during monsoon season and H/L ratios during hot and dry season, but we found no pattern in oxidative stress (ROM and SOD) levels. Hot season also associated with a decline in body weight. The present study shows how different physiological parameters (FCM levels and H/L ratio), molecular (oxidative stress) and body condition vary with seasonal changes, and how these parameters might allow individuals to adapt to such variations. Our results on an endangered long-lived species are crucial in indicating the most productive timing for conservation efforts, predicting how individuals cope with environmental changes, and allow for a more accurate representation of how animal physiology operates in nature.

## Teaser

Seasonality is an important driver of variation in animal life-history traits such as growth, survival and reproduction, but the underlying physiological mechanisms responsible for such variation remain unknown, we charcterized the seasonality of two stress markers (FCM & H/L ratio), two oxidate stress markers (ROM & SOD) and body weight in a long-lived mammal to investigate the physiological consequences of seasonally induced environmental change.

## Introduction

Environmental conditions play a fundamental role in shaping individual life history trajectories and consequently regulate populations over time (Southwood, 1988). The annual life cycle of many species has adapted to seasonality and the oscillating productive or unproductive periods with necessary trade-offs in life history traits (Varpe, 2017). This way, seasonal changes within both biotic and abiotic parameters are responsible for driving births (Henderson *et al.,* 2014), survival (Tecot, 2010), body condition (Ejsmond *et al.,* 2018) and overall fitness (Harrison *et al.,* 2011; Buckley *et al.,* 2017).

Environmental factors vary along the year and entail a range of consequences for animals’ life history. Seasonality is responsible for changes in weather patterns like temperature and rainfall, influencing food availability (Jahn *et al.,* 2010; Chang *et al.,* 2014), predation and social behaviour (Pruitt *et al.,* 2011; Kiffner *et al.,* 2014) or even disease patterns (George *et al.,* 2011; Cizauskas *et al.,* 2015). Changing abiotic environmental measures can have drastic effects on individuals. For example, changes in temperature, precipitation and snow depths can reduce food availability (Kitaysky *et al.,* 2007), disrupt the social environment (Creel *et al.,* 2013), and affect predator–prey interactions (Hirschenhauser *et al.,* 2000).

While previous studies have shown that seasonality has an important effect on the animal life history traits, less is known about the response of underlying physiological mechanisms in animals living in natural habitats (but see Romero, 2002; Wingfield, 2008). Thus, to achieve a complete understanding of the impact and consequences of these seasonal effects on the animals’ life history, it is necessary to study the physiological mechanisms. A key mechanism known to mediate the individuals’ response to changes in environmental conditions is the hyperactivation of the hypothalamic–pituitary–adrenal (HPA) axis leading to increased glucocorticoid release in the bloodstream (Reeder and Kramer, 2005). Glucocorticoids are central mediators that respond to challenges and regulate metabolism (Sapolsky *et al.,* 2000; Opata, 2016), with important consequences to individual fitness components such as reproductive success or survival (Vitousek *et al.,* 2019; Schoenle *et al.,* 2020) or physiological outcomes such as immune response (Dickmeis *et al.,* 2013). Individuals living in natural conditions are directly affected by changing weather, especially temperature, precipitation and food availability as part of their environmental seasonality, which can lead to an increase in glucocorticoids (Jessop *et al.,* 2016; de Bruijn and Romero, 2018). In addition to glucocorticoids, the ratio between heterophils or neutrophils to lymphocytes (H/L ratio) in blood smears can also be used to assess stress in animals. Studies suggest that this haematological response to stress is consistent across the taxa (Davis *et al.,* 2008), and shows consistent variation in seasonal cycles (Owen and Moore, 2006; Carbillet *et al.,* 2019; Seltmann *et al.,* 2020).

Acute or prolonged HPA axis activation, resulting from acute or chronic stress, involves a high diversity of metabolic and physiological effects that result in elevated oxidative stress, thereby providing a possible mechanism for the costs of stress (Costantini *et al.,* 2011; Majer *et al.,* 2019). Oxidative stress can arise from the reactive oxygen species (ROS) generated from exogenous sources (UV radiation and pollutants), but the majority of intracellular ROS are thought to arise as a by-product of aerobic metabolism and ATP production in the mitochondria (Balaban *et al.,* 2005). ROS are highly reactive and will cause oxidative damage to various biomolecules (often estimated from reactive oxygen metabolites, ROMs). Such damage can either be prevented by defence mechanisms known as antioxidant defences or repaired in some cases after they occur. Oxidative stress is thus the result of an imbalance between antioxidant defences and ROS production (Sies *et al.,* 2017). Especially in the case of chronic stress, oxidative stress can account for the detrimental effects of the prolonged action of the glucocorticoids (Costantini *et al.,* 2011) making it an important marker to account for when evaluating physiological consequences of stress. Studies on several species now show that such oxidative stress biomarkers can vary in seasonal environments (Chainy *et al.,* 2016; Marasco *et al.,* 2017). Another biomarker underlying resilience to stress in different seasons is the body weight, given it represents the nutritional reserves accumulated in each season (Dugdale *et al.,* 2011; Kroeger *et al.,* 2018). Consequently, seasonal variation in body weight is often linked to food availability (Dugdale *et al.,* 2011; Mumby *et al.,* 2015b). However, few studies have investigated the multiple different measures of stress in animal populations living in their natural environment and compared their responses to seasonal variation.

**Figure 1:**
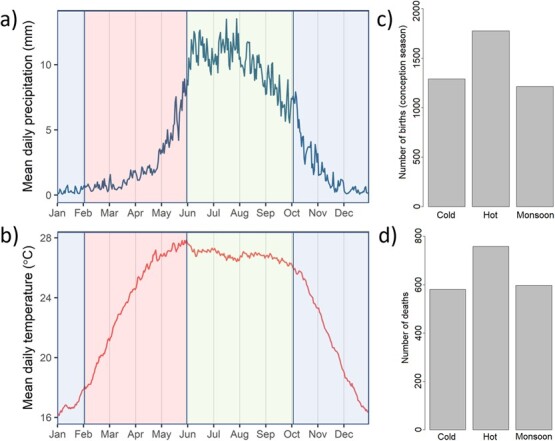
Description of seasonality. (a) The mean daily temperature and (b) precipitation across years (1951–2007 or 1961–2007) from the deciduous forest region of Sagaing and Kachin divisions and seasonal number of (c) observed births and (d) observed deaths in Myanmar timber elephant population (5843 individuals from 1960–2018). The environmental data was obtained from the APHRODITE (Asian Precipitation - Highly Resolved Observational Data Integration Towards Evaluation) daily gridded dataset and was restricted using data from MIMU (Myanmar Information Management Unit - A unit of the UN in Myanmar (Yatagai *et al.,* 2012)).

In this study, we aim to identify the effects of seasonal climate fluctuation on the physiology of a species using a range of molecular and physiological markers of stress in Asian elephants (*Elephas maximus*). The unique semi-captive nature of the Asian elephant population in Myanmar offers several key advantages by including repeated samples from known individuals that allows us to measure HPA function over different time windows alongside other health and demographic measures, in order to obtain an integrative measure of stress across seasons and years whilst controlling for several key confounding variables. Furthermore, the exceptional lifespan of up to 80 years of Asian elephants (Jackson *et al.,* 2020) makes them a uniquely long-lived model species to observe the accumulation and long-term effects of stress on physiology, reproduction and survival rates down the line. In this study, we sampled individuals three times a year (once per season) from 2011 to 2018. We expect our measures of stress to reflect seasonally varying climatic conditions, food availability and health (Franco dos Santos *et al.,* 2020) that could also have downstream effects to oxidative balance and body condition. In particular, we expect to observe signs of stress during hot season, which has the highest seasonal Asian elephant mortality in Myanmar (Mumby *et al.,* 2015a and b, Fig. 1d), likely at least in part due to the highest annual temperatures and decreased vegetation from lack of rain.

## Methods

### Study population and site

The elephant populations in our study live primarily in Katha and Kawlin logging camps, located in Sagaing region in north-western Myanmar. The climate in Myanmar is subtropical and tropical monsoon climate with three distinct seasons: cold (in October, November, December and January), hot (in February, March, April and May) and monsoon (in June, July, August, September) (Fig. 1). The seasons are classified according to precipitation (Fig. 1a) and temperature (Fig. 1b) data in the following way: the cold and dry season (blue), the hot and dry season (red), the monsoon season (light green). These seasons are not as stationary as presented here and vary slightly in length each year (Romatschke and Houze Jr, 2011). Food abundance is the lowest during hot season in spring, peaks during monsoon season and stays moderate during cold season. Elephants are generally aseasonal breeders and are able to reproduce throughout the year but we found that in our population birth rate varied considerably between the different months of the year, being highest in hot season and lowest in monsoon (Fig. 1c)**.** Similarly, mortality varies seasonally with the lowest mortality rate occurring during monsoon and cold seasons compared to relatively higher rates in hot season (Mumby *et al.,* 2015b; Fig. 1d).

The present study was conducted on government-owned Myanmar timber elephants that inhabit forest camps, distributed across Myanmar. The government-owned elephants are used during the day as riding, transport and draft animals, which they do under supervision of a personal human caretaker (“mahout”) (Crawley *et al.,* 2019). The Myanma Timber Enterprise (MTE) imposes regulations on workload, travel distance, and working period for all elephants (Zaw, 1997; Mar, 2002), but our study focussed for logistic reasons on animals not involved with work-tasks at the time of the study. Once calves turn 4 or 5, they are separated and begin light training. Around the age of 20 the elephants are considered “adults” until they become “seniors” from about the age of 50 onwards.

The working population is composed of both captive-born and wild-captured individuals as the workforce was traditionally supplemented by capturing elephants from the wild (Jackson *et al.,* 2019). The survival and reproductive rates of such individuals in captivity are lower than those of captive-born elephants (Lahdenperä *et al.,* 2018; Lahdenperä *et al.,* 2019). Capturing wild elephants was largely banned in 1990s, and as a consequence, most wild-born individuals included in our study are relatively old and have lived in captivity for a long period. We control for such variation in origin and early conditions in our analyses (see below).

Although elephants are managed as draft and transport animals by the MTE, they are more frequently described as “semi-captive” and live largely under natural conditions: (1) Elephants are released to the forest at night to forage naturally and interact with conspecifics, and food supplementation has historically been minimal; (2) Breeding rates are natural with matings and births occurring in forests at night, and with no reproductive management of the population; (3) Timber elephants are never culled and numbers are not restricted or managed; and (4) Elephants benefit from bimonthly veterinary checks, with trained MTE veterinarians being responsible for the basic upkeep of the elephants and predominantly treating wounds and other injuries. At the same time, demographic, life history, health and mother lineage information have continuously been recorded in logbooks for over a century by MTE vets. Thanks to these logbooks we know, for each elephant, its unique registration number (ID); name; sex; camp; date of birth; origin (captive-born or wild-caught); age; age of capture (for wild-caught); weight; alive or dead status; mother’s ID number; last seen- date. In our study we only used samples from calves and adult elephants used to human handling.

### Dependent variables: stress, oxidative damage, antioxidant defence and health

In our study, we analysed the seasonal variation in five physiological parameters that described the stress, oxidative damage, antioxidant defence and health of the elephants. We used body weight as a proxy of the health status, FCMs and H/L ratio represented stress markers, ROMs as a proxy for oxidative damage and SOD as a proxy for antioxidant defence. The elephants were measured and sampled in the mornings, with all of our study animals, for logistic reasons, not involved in work tasks at all during the study. Samples were collected and kept in cooling boxes a maximum of 12 hours before processing. The blood was centrifuged, separated into small 2 ml tubes and frozen while faecal samples were directly frozen after collection.

#### Do FCMs vary with season?

Short-term stress levels from the past day were measured from FCMs (Wingfield and Romero, 2015). Faecal samples were collected every month immediately after defecation on the same day for each individual in Katha and Kawlin logging camps between January 2013 and May 2018. The samples were frozen at −20°C until dried at 50°C for 12 hours and analysed at the Veterinary Diagnostic Laboratory in Chiang Mai, Thailand (Watson *et al.,* 2013; Crawley *et al.,* 2021). FCMs were extracted from samples using a protocol for boiling extraction (Crawley *et al.,* 2021) and concentrations were assessed using double antibody enzyme immunoassay (EIA) (Watson *et al.,* 2013; Brown *et al.,* 2019), where for achieving consistent results the original EIA protocol was performed by using Nunc MaxiSorp® plates, room temperature substrate reagents and dark incubation conditions. Samples were analysed in the lab in five batches between years 2015–2018, where fresh reagents were made and used for each batch. Batch effect was included as a random factor and controlled for in all analyses (see description below). The intra-assay variation was <10% (samples with duplicate intra-assay coefficients of variability >10% were reanalysed) and assay sensitivity was 0.099 ng/g faeces. We collected 1851 samples of FCMs from 261 individuals aged 4–69 years (Table A1, Table 1).

**Table 1:** Model selection testing the seasonal effect on health- and stress-related parameters using mixed models

**Trait**		**K**	**AICc**	**ΔAICc**	**AICcWt**
**FCMs**	*Base: FCM ~ F(sex) + F(age) + F(camp) + F(origin) + F(birth season) + R(1|year/ID)*				
(1851 measures;	Base + Season	16	23520.70	0.00	1
261 individuals)	Base	14	23592.33	71.63	0
**H/L Ratio**	*Base: HL ~ F(sex) + F(age) + F(camp) + F(origin) + F(birth season) + R(1| year/ID)*				
(562 measures;	Base + Season	15	666.90	0.00	1
181 individuals)	Base	13	695.29	28.39	0
**ROMs**	*Base: ROMs ~ F(sex) + F(age) + F(camp) + F(origin) + F(birth season) + R(1|ID) + R(1|year)*				
(354 measures;	Base	12	3376.11	0.00	0.77
106 individuals)	Base + Season	14	3378.48	2.38	0.23
**SOD**	*Base: SOD ~ F(sex) + F(age) + F(camp) + F(origin) + F(birth season) + R(1|year/ID)*				
(619 measures;	Base	13	9456.16	0.00	0.64
118 individuals)	Base + Season	15	9457.31	1.15	0.36
**Standardised weight**	*Base: SW ~ F(sex) + F(age) + F(camp) + F(origin) + F(birth season) + R(1|year/ID)*				
(5039 measures;	Base + Season	18	64621.61	0.00	1
481 individuals)	Base	16	64634.49	12.88	0

We performed a model selection per trait. Base model contains fixed (F()) and random (R()) confounding variables. FCMs and H/L ratio are proxies of the stress level. ROMs represent oxidative stress and SOD represents the antioxidant defence. Standardised weight is a proxy of the health status. We used gaussian distributions for all traits and used a log-link for FCMs, H/L ratio and SOD. For each model selection, we provide the number of parameters (K), the corrected AIC (AICc), the difference in AIC between the focal and the best fitting models (ΔAICc), the AIC weight (AICcWt). The best fitting model is highlighted in grey.

#### Does H/L ratio vary with season?

We assessed stress integrated over long-term by measuring H/L ratios, using blood samples collected at least three times per year (once during each season) from March 2016 to April 2018 in Kawlin and Katha logging camps. The blood was taken from an ear vein and collected in an EDTA tube, which was refrigerated before analysis in the laboratory. We performed a blood smear stained with Romanowsky stain and counted differential leucocyte cells manually. For each slide, we identified the 100 first leucocytes (monocytes, lymphocytes, heterophils, eosinophils, basophils): from here we were able to determine the heterophils/lymphocytes ratio. We determined 562 H/L ratios from 181 individuals aged 4–69 years (Table A1, Table 1).

#### Do ROMs vary with season?

We assessed physiological consequences of stress by estimating the oxidative damage (ROMs) in the plasma once during each season from April 2014 to April 2018. To do so, we collected blood from an ear vein and stored it in serum separator tubes that were refrigerated before analysis in the laboratory. The plasma was collected, frozen and used to perform d-ROM tests (5 μL of plasma, Diacron International, Italy) following the manufacturer instructions. The d-ROMs test measures the concentration of hydroperoxide (ROOH) as ROMs. The concentration of ROOH is a marker of a potential exposure to oxidative stress and is thus a proxy of oxidative damage. ROMs concentrations were expressed as mg of H_2_O_2_ equivalent/dL. The d-ROMs test has been largely validated in birds and mammals (Costantini, 2016). We analysed 354 repeated samples from 106 individuals aged 4–65 (Table A1, Table 1). Intra-individual variation among these samples based on the duplicates was 5.12 ± 1.02%**.**

**Figure 2:**
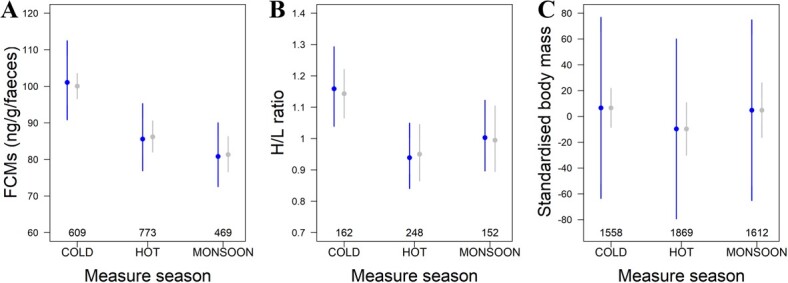
We showed seasonal variation in FCMs level (A), heterophil and lymphocyte ratio (H/L ratio, B) and standardised body weight (C). Figures 2A–C present predicted values (blue dots) from the linear mixed models (Table A2) with SE bars and observed means (grey dots) with [95]CI error bars, and the number of repeated measurements for each season.

#### Do SOD vary with season?

We assessed the enzymatic activity of the antioxidant enzyme SOD present in the plasma (see *ROMs* section for details concerning the collection of plasma samples) once per season from April 2014 to April 2018. SOD is one of the first enzymes in the antioxidant defence cascade, where the dismutation of superoxide radical is catalysed into oxygen and hydrogen peroxide. We used the SOD activity kit (Enzo® Life Sciences, USA) on the plasma following the manufacturer instructions. The test measures *in vitro* the kinetics of inhibition in the superoxidase production due to SOD antioxidant activity. SOD activity is presented as U (units of enzymatic activity) mL^−1^. Our dataset had 619 repeated samples from 118 individuals aged 4–65 (Table A1, Table 1). Intra-individual variation based on the duplicates was 23.3 ± 4.64%**.**

#### Does body weight vary with season?

Finally, we investigated a seasonal effect on body weight. We collected body weight between July 2011 and November 2018 mostly in Katha and Kawlin camps (88% of the measures). The other logging camps were located in elsewhere in Sagaing, Mandalay/Magway, Kachin, Shan, Naypyidaw and Bago. An Eziweigh 3000® scale, capable of weighing up to 9000 kg to the nearest 10 kg, was used to weigh elephants. As individual's age is linked to its size, we investigated seasonal variation in standardised body weight. To do so, we modelled age-specific variation in body weight using a Bertalanffy growth curve shown to be the best fit in our population (Mumby *et al.,* 2015b). As performed in Mumby *et al.,* (2015b), we ran separate curves for males and females. We used residuals of sex-specific fits as the standardised body weights. We had 5039 measures from 481 individuals aged 0–72, where a majority were juvenile elephants (Table A1, Table 1).

### Statistical analyses

To investigate the effects of season on the health status, stress markers, oxidative damage and antioxidant defence parameters, we used generalised linear mixed modelling with a Gaussian family and a log-link function for FCMs, H/L ratio and SOD (*lme4* package (Bates *et al.,* 2015)) and linear mixed modelling with a Gaussian family for ROMs and standardised body weight data, with R version 3.5.2 (R Core Team, 2020). For all models, we included the season when the elephants were weighted or sampled as a fixed 3-level factor (cold, hot, monsoon; Franco dos Santos *et al.,* 2020) and four fixed confounding variables: sex (2-level factor; male and female; Franco dos Santos *et al.,* 2020), origin (2-level factor, wild-caught and captive-born; Lahdenperä *et al.,* 2019), age (4-level factor; calves (0–10 or 4–10 y.o.), juveniles (10–20 y.o.), adults (20–54 y.o) and retired (54+); Franco dos Santos *et al.,* 2020) and the logging camp as a two-level factor in all models except for body weight analysis for which we had the logging camps as a nine-level factor. We included birth season only for captive elephants for which we knew the exact birth date. Although we did not find a significant impact of birth season on any stress marker, oxidative stress markers or body weight in our models, it was included in the base models as birth season can have long-term effects on elephant health (Mumby *et al.,* 2015a). To do so, we included an interaction with no main effect between the birth season and the origin of the individual (coded 0 for wild and 1 for captive elephants). To account for the repeated measures and yearly fluctuations in the data (Jackson *et al.,* 2020), we included the identity of individuals and the year of collection as random intercepts in all models. We nested random effects (year/ID) for all traits except for ROMs because of singularity issues. We back-transformed the results when the log-link function applied and provided exp(β)[exp(β-SE); exp(β + SE)].

#### Akaike information criterion-based model selection

For each parameter, we compared two models: (1) a base model with sex, age, origin, camp and birth season as fixed factors and (2) the base model (1) including season (3-level: hot/cold/monsoon) as a fixed factor (Table 1). The most competitive models were selected using the Akaike information criterion (AIC), considering each random effect as one parameter (Pinheiro and Bates, 2002). Following the principle of parsimony, the model with the lowest AIC was retained as the best model. Where the difference in AIC between competing models was less than two, we retained the simplest model (Burnham and Anderson, 2002). We also calculated the Akaike weight (AICw) for each model to provide the relative likelihood that the model was the best among the candidate models. The model comparisons were carried out using maximum likelihood (ML) approach, but the results for the best models are reported using restricted maximum likelihood (REML) estimations. Only the results for the best models selected as described above are reported.

## Results

### Seasonal variation in the stress markers using FCMs

FCMs varied between 0.42 and 370.72 ng/g/faeces in our sample. The results, which accounted for within-individual, temporal and spatial variations, confirmed that elephants showed the highest level of FCMs in cold season (original scale coefficients for cold season: 101.07, SE[90.85;112.44] ng/g/faeces, hot season: 85.59, SE[76.90;95.26] ng/g/faeces, monsoon season: 80.82, SE[72.57;90.01] ng/g/faeces; Table 1; Table A2; Fig. 2A). Elephants sampled in hot season showed higher FCMs values than the ones sampled during monsoon (β_hot-monsoon_(log-link scale) = 1.16 ± 0.030). Overall, FCM level decreased by 20% between cold and monsoon seasons. Our statistical analyses supported a strong seasonal effect on FCMs (Table 1) and that FCM levels were higher in males (β_male_(log-link scale) = 0.08 ± 0.03, Table A2), but we found no difference in FCM levels between captive- and wild-born individuals or according to their age (Table A2).

### Seasonal variation in stress markers using H/L ratio

H/L ratio varied between 0.20 and 3.45. Here, we found that H/L ratio showed the highest level in cold season and the lowest was found in hot season (Table 1; Table A2; Fig. 2B). On average, elephants displayed an H/L ratio of 1.16 SE[1.04;1.29] in cold season, 0.94 SE[0.84;1.05] in hot season and 1.00 SE[0.90;1.12] during monsoon season. Elephants sampled during monsoon showed higher H/L ratio values than the ones sampled in hot season (β_hot-monsoon_(log-link scale) = 0.89 ± 0.04, as the difference in the mean response did not overlap with 0). Overall, H/L ratio decreased by 22% between cold and hot seasons. Our statistical analyses adjusting for sex, age, camp, birth season and origin (captive/wild-born) supported a significant seasonal effect on H/L ratio (Table 1) but we observed no difference in H/L ratios between age groups or the sexes (Table A2).

### No seasonal variation in oxidative stress markers using ROMs

ROMs varied between 3.10 and 35.88 mg H_2_O_2_/dL. On average, adult elephants had significantly lower ROM concentrations compared to the other age groups (β_adult_ = 16.35 ± 0.88, Table A2), but even after accounting for within-individual, temporal and spatial variation, we did not detect any seasonal effect on ROMs between cold season (16.54 ± 5.95 mg H_2_O_2_/dL**)**, hot season (17.11 ± 6.56 mg H_2_O_2_/dL**)** and monsoon season (17.11 ± 7.19 mg H_2_O_2_/dL; cold in intercept, β_hot_ = −0.50 ± 0.60, β_monsoon_ = 0.28 ± 0.61 Table 1). We did not observe any differences between the sexes (Table A2).

### No seasonal variation in antioxidant defence using SOD

SOD varied between 15.4 and 498.5 U/mL. We did not detect any seasonal effect on SOD (in cold season 136.46 SE[121.73;153.04] U/mL, hot season 144.84 SE[129.67;161.85] U/mL and monsoon season 142.51 SE[127.55;159.28] U/mL Table 1) nor any significant differences between the sexes or age groups (Table A2).

### Seasonal variation using body weight

Body weight varied between 182 and 4582 kg across all animals included in our sample. After accounting for within-individual, temporal and spatial variation, we detected a seasonal effect on standardised body weight (cold in intercept, β_hot_ = −6.78 ± 4.02, β_monsoon_ = 3.88 ± 3.93; Fig. 2C; Table 1 for the model selection; Table A2 for the estimates of the final model). Elephants showed a decrease of 3.8% of their standardised weight during hot season compared to cold season.

## Discussion

This study investigated seasonal variation in physiological markers of stress in Asian elephants living in their natural environment in Myanmar. Our results provide evidence of seasonal variation in stress markers (FCM and H/L ratio), where on average FCMs and H/L ratios peaked during cold season. The lowest concentrations were measured in monsoon season for FCM and hot season for H/L ratio. We also found seasonal fluctuations in body weight. In contrast, we observed no significant seasonal changes in a marker of oxidative damage (ROM) or in a marker of antioxidant enzyme activity (SOD). These results suggest that seasonal variation can affect elephant stress physiology and body condition, but the effects at the molecular level appear more complex.

Our results indicate seasonal variation in FCM and H/L ratio levels and found that FCM concentrations had a larger range than previously measured in elephants ([20;80ng/g/feces], Foley *et al.,* 2001) while H/L ratios were within normal range of captive elephants (Davis *et al.,* 2008). There are multiple hypotheses for the implications of these results. First, seasonal variation in FCM and H/L ratio could reflect the seasonal changes in temperature and climate. High temperature has been linked to the highest stress marker concentrations in both FCM and H/L ratio in birds (Al-Murrani *et al.,* 1997; Akşit *et al.,* 2006), in cattle (Maibam *et al.,* 2014) and in goats (Banerjee *et al.,* 2015), but these observations are not in line with our results. In Myanmar, the highest temperatures occur during hot and monsoon seasons, but instead we observed the highest FCM and H/L ratios during the coldest season. Second, FCM has a strong link to metabolism and energy regulation that can reflect seasonal variation in food availability and thermoregulation (Kitaysky *et al.,* 2007). This hypothesis could explain low levels of FCM during monsoon season when food abundance is the highest. However, we should then observe a gradual increase in FCM from cold to hot season as food abundance decreases, but instead FCM levels peak during cold season. Third, our data has a bias towards juvenile calves who are in taming and training. Taming is a highly stressful process for calves organised during the cold season in November/December that has been linked to increased calf mortality (Crawley *et al.,* 2020). Our data does not include samples from juveniles or their mothers during taming and the peak we observed in FCMs and H/L ratios during cold season did not depend on age, which indicates a more general response to environmental changes. To be sure, we reran our FCM results without calves and juveniles, which did not change our results (Table A3, Fig. A1). Seasonal variation in stress levels can also predispose individuals to infections and parasites, however, the interaction between these stress markers such as FCM and H/L ratio and parasites is complex (Pedersen and Greives, 2008; Hofmeester *et al.,* 2019): in mice FCM levels increased in infected individuals only during a food shortage. In addition, a study on Bornean elephants found seasonal variation in trematode, cestode and nematode load, where the seasonal peak in parasite prevalence was different for each parasite species (Hing *et al.,* 2013). Interestingly, in our study population, the number of conceptions is the highest but survival is the lowest in the following hot season (Fig. 1c,d), which is the only season the elephants get a break from work and can forage freely but also a physically challenging time of the year due to high temperatures and low food abundance. While this trend is likely a consequence of elephants’ annual working schedule, where the holidays are set during the most physically challenging time of the year, high FCM and H/L ratio levels during cold season could even be linked to a lower chance of survival in the following season.

We did not observe an association between oxidative damage (ROM) or activity of an antioxidant enzyme (SOD) and seasonality. The models showed high inter-individual variation in both markers, which could indicate more complex mechanisms in the oxidative stress response. Stressful environments may accumulate oxidative damage in cells slowly over time, causing a lag. Hence, rather than observing a direct impact within one season, in a long-lived mammal such as the Asian elephant seasonal oxidative damage may be delayed and cumulative throughout the animal’s life leading to gradual cellular senescence that has an impact on health later in life rather than within a year (Herbig *et al.,* 2006; Campisi and d’Adda di Fagagna, 2007). However, because a big portion of our study population consists of juvenile elephants (Table A1), it could be more difficult to observe these patterns. We also may have missed seasonal changes in other antioxidant defence markers (enzymatic or non-enzymatic) (Belló-Klein *et al.,* 2000; Megahed *et al.,* 2008; Bonda-Ostaszewska *et al.,* 2012). In the case of SOD, seasonal fluctuations may have been lost in part due to high uncertainty in our measurements. Although the choice of dROM as a marker has been validated in other mammals (Costantini, 2016), we must note that it may lack of specificity or have interference with other serum or plasma components, which may have contributed to masking biological signals.

Finally, we found seasonal effect on elephant body weight that supported findings of an earlier, much smaller study on Myanmar timber elephants, which also found the lowest body weights during hot season (Mumby *et al.,* 2015b). The key characteristics of hot season are high temperatures, low precipitation and low food abundance. While all these factors could be linked to the decline in body weight, food availability is the most likely determinant of body weight and can affect birth rates and survival of the population (Kitaysky *et al.,* 1999; McEwen and Wingfield, 2003; Monaghan, 2014). Thus, these results likely, in part at least, reflect the physiological reaction to the decrease in food abundance during hot season, which also could vary annually in severity of its impact on body weight.

Our study has several strengths and weaknesses. One issue with studies on natural populations attempting to measure chronic stress based on glucocorticoid metabolites is that the stressor in our study is unknown and/or complex in origin and thus the interpretation of the results becomes challenging since we cannot identify the starting point and sometimes even the source or features of the stress or measure time passed after the stressful event. Chronic stress is capable of both increasing and decreasing HPA activity depending on features of the stress and time passed after the stressful event (Miller *et al.,* 2007). In humans, chronic stress first begins with a peak in cortisol secretion, but over time after the stressor is no longer present and the HPA axis activity decreases, cortisol concentration rebounds to below the baseline (Miller *et al.,* 2007). Although some stressors are predictable, such as seasonal changes in food availability and temperature, there may be unpredictable aspects within these seasonal changes we are not aware of (e.g. amplitude and/or frequency of weather anomalies or food availability). Second, as especially FCM concentrations can produce inconsistent results, it has been suggested that body weight should also be included as a stress indicator in studies on physiological stress, because it produces more reliable results and is a direct proxy of the animal’s health (Dickens and Romero, 2013). To avoid relying solely on one stress marker, we included several stress markers (FCM and H/L ratio) to evaluate their potential impacts on elephant body condition and cellular stress markers. However, in our data the sampling periods of these markers do not overlap fully, which leaves room for uncertainty of the results where the sampling periods do not match. Third, the mechanistic validity and utility of growth curves in mature animals has been debated as predictive curves such as Bertalanffy growth curve do not consider reproduction (Day and Taylor, 1997; Marshall and White, 2019). However, most of our data consists of calves and juvenile elephants, which have not yet reached maturity.

Understanding seasonal variation in stress physiology has conservation value, because such seasonal variation can cascade and influence mortality and fertility rates across the year (Fig. 1; Mumby *et al.,* 2015a and b). In wild African elephants in Amboseli, Kenya, drought years were associated with higher calf mortality (Moss, 2001; Foley *et al.,* 2008), but little is known of the physiological mechanisms for such within and between year differences in vital rates to-date, hindering opportunities for intervention. Our results show a complex pattern of the long-term effects of season in long-lived species and provide a good basis for further studies about the underlying physiology of seasonal variation in life history and vital rates. We found signs of long-term effects of stress on a long-lived species threatened by poaching, habitat loss and human-elephant conflict. We thus recommend intensifying and targeting health checks on stress markers during cold season. Unsurprisingly, our results suggested that seasonal changes in environmental conditions such as high temperatures and food availability have an impact on the elephant body condition, which should be considered in the working schedules of these timber elephants, and noting that later adjustments may be needed due to climate change. Our results also suggest that FCM and H/L ratio are good markers for monitoring physiological response to environmental variation. These results are of broad general importance because they show how seasonality can influence stress physiology and body condition in long-lived animals that typically live over several of such seasonal cycles in nature. However, further investigation of other ageing or stress markers, for example telomeres as an ageing marker or mitochondrial function as a metabolic marker, is needed to explore the long-term effects of seasonal impact on physiology.

## Funding

This work was supported by the University of Turku Graduate School, the Academy of Finland [grant numbers 292368 and 324257], the European Research Council [grant number CoG 648766], the Marie Curie fellowship scheme [grant number 659937-AGEISM], the Turku Collegium for Science, Medicine and Technology and the Finnish Cultural Foundation.

## Conflicts of interest statement

The authors have no conflicts of interest to declare.

## Data availability

The data set is available from the corresponding author.

## Author contributions

S.U.: Methodology, data curation, analysis, visualization, writing (original draft). V.B.: Conceptualization, methodology, analysis, visualization, writing (reviews). D.S.: Resources, data curation, writing (reviews). W.H., H.H.A., U.K.N.: Resources, data curation. S.R.: Conceptualization, data curation, supervision, writing (reviews). V.L.: Conceptualization, supervision, writing (reviews).

## Supplementary Material

Web_Material_coad029
