# Supplementary material for: Seasonal variation in molecular and physiological stress markers in Asian elephants
Source: Conserv Physiol. 2023 May 19;11(1):coad029. doi: 10.1093/conphys/coad029 (PMC10660384; doi:10.1093/conphys/coad029)
Supplement: Web_Material_coad029 [file web_material_coad029.pdf]

# Supplementary material

## Seasonal variation in molecular and physiological stress markers in Asian elephants

Susanna Ukonaho[1*], Vérane Berger[1*], Diogo J. Franco dos Santos[1], Win Htut[2], Htoo Htoo Aung[2], U Kyaw Nyeing[2], Sophie Reichert[1#] and Virpi Lummaa[1#]

* shared first authors, equal contributions

# shared last authors, equal contributions

[1] Department of Biology, University of Turku, FIN-20014 Turku, Finland

[2] Myanma Timber Enterprise, MONREC, Myanmar

**Table A1**. The number of measures for each response per age and sex.

| | Age category | Female | Male |
|---|---|---|---|
| **FCMs** | **Calves** | 344 | 457 |
| | **Juveniles** | 265 | 217 |
| | **Adults** | 320 | 110 |
| | **Seniors** | 99 | 39 |
| **H/L ratio** | **Calves** | 80 | 103 |
| | **Juveniles** | 98 | 69 |
| | **Adults** | 114 | 36 |
| | **Seniors** | 51 | 11 |
| **ROMs** | **Calves** | 109 | 152 |
| | **Juveniles** | 102 | 62 |
| | **Adults** | 52 | 31 |
| | **Seniors** | 24 | 2 |
| **SOD** | **Calves** | 116 | 173 |
| | **Juveniles** | 114 | 71 |

| | | | |
|---|---|---|---|
| | **Adults** | 72 | 38 |
| | **Seniors** | 31 | 4 |
| **Standardised weight** | **Calves** | 834 | 1134 |
| | **Juveniles** | 731 | 590 |
| | **Adults** | 926 | 481 |
| | **Seniors** | 261 | 82 |

**Table A2.** Output of the retained models by the model selection (see Table 1). We provide the estimates, SE, P-value. Standardised body weights are residuals of the age-specific variation in body weight. T-values are in bold.

| **Dependent variable** | | **Estimate** | **SE** | **T-** or P-value |
|---|---|---|---|---|
| **FCMs** | Intercept | 4.34 | 0.13 | <0.01 |
| Log-link | $Season_{Hot}$ | -0.08 | 0.02 | 0.00 |
| | $Season_{Monsoon}$ | -0.23 | 0.03 | <0.01 |
| | $Origin_{captive}$ | 0.10 | 0.05 | 0.07 |
| | $Sex_{male}$ | 0.08 | 0.03 | 0.00 |
| | $Age_{Calves}$ | -0.09 | 0.06 | 0.14 |
| | $Age_{Juveniles}$ | -0.09 | 0.06 | 0.12 |
| | $Age_{Adults}$ | 0.02 | 0.06 | 0.72 |
| | $Camp_{Katha}$ | 0.05 | 0.07 | 0.47 |
| | $Camp_{Kawlin}$ | 0.03 | 0.07 | 0.68 |
| | $Camp_{West\ Katha}$ | -0.40 | 0.11 | 0.00 |
| | $Origin_{captive}$ : $Birthseason_{Hot}$ | 0.01 | 0.04 | 0.86 |
| | $Origin_{captive}$ : $Birthseason_{Monsoon}$ | -0.07 | 0.04 | 0.06 |
| **HL ratio** | Intercept | -0.04 | 0.12 | 0.72 |
| Log-link | $Season_{hot}$ | -0.24 | 0.04 | 0.00 |
| | $Season_{monsoon}$ | -0.13 | 0.04 | 0.00 |
| | $Origin_{captive}$ | -0.02 | 0.10 | 0.84 |
| | $Sex_{male}$ | 0.06 | 0.06 | 0.29 |
| | $Camp_{Kawlin}$ | 0.18 | 0.07 | 0.01 |
| | $Camp_{West\ Katha}$ | -0.10 | 0.10 | 0.34 |
| | $Age_{Calves}$ | -0.08 | 0.12 | 0.48 |
| | $Age_{Juveniles}$ | -0.05 | 0.11 | 0.69 |
| | $Age_{Adults}$ | 0.01 | 0.10 | 0.92 |
| | $Origin_{captive}$:$Birthseason_{hot}$ | 0.02 | 0.08 | 0.80 |
| | $Origin_{captive}$:$Birthseason_{monsoon}$ | -0.01 | 0.08 | 0.90 |
| **ROMs** | Intercept | 18.24 | 2.80 | **6.52** |
| | $Origin_{Captive}$ | -2.08 | 1.21 | **-1.72** |
| | $Age_{Juveniles}$ | -0.53 | 0.60 | **-0.88** |
| | $Age_{Adults}$ | -1.89 | 0.88 | **-2.15** |
| | $Age_{Seniors}$ | -0.08 | 1.21 | **-0.07** |
| | $Sex_{M}$ | 0.43 | 0.50 | **0.87** |
| | $Camp_{Kawlin}$ | -0.70 | 1.82 | **-0.39** |
| | $Camp_{West\ Katha}$ | -2.36 | 2.27 | **-1.04** |
| | $Origin_{Captive}$:$Birthseason_{Hot}$ | 0.09 | 0.67 | **0.14** |
| | $Origin_{Captive}$:$Birthseason_{Monsoon}$ | 0.43 | 0.64 | **0.67** |
| **SOD** | Intercept | 5.07 | 0.20 | <0.01 |
| Log-link | $Origin_{Captive}$ | -0.05 | 0.12 | 0.70 |
| | $Age_{Juveniles}$ | -0.04 | 0.07 | 0.53 |
| | $Age_{Adults}$ | 0.07 | 0.09 | 0.43 |
| | $Age_{Seniors}$ | 0.12 | 0.13 | 0.32 |
| | $Sex_{M}$ | -0.10 | 0.06 | 0.06 |
| | $Camp_{Kawlin}$ | -0.19 | 0.16 | 0.22 |

| | | | | |
|---|---|---|---|---|
| | $Camp_{West\ Katha}$ | 0.05 | 0.21 | 0.80 |
| | $Origin_{Captive}$:$Birthseason_{Hot}$ | 0.07 | 0.08 | 0.33 |
| | $Origin_{Captive}$:$Birthseason_{Monsoon}$ | -0.07 | 0.07 | 0.35 |
| **Standardised body weight** | Intercept | 179.50 | 35.99 | **4.99** |
| | $Season_{hot}$ | -6.78 | 4.02 | **-1.69** |
| | $Season_{Monsoon}$ | 3.88 | 3.93 | **0.99** |
| | $Origin_{captive}$ | -15.29 | 26.07 | **-0.59** |
| | $Sex_{male}$ | -50.51 | 17.12 | **-2.95** |
| | $Camp_{Kachin}$ | -168.03 | 109.28 | **-1.54** |
| | $Camp_{Katha}$ | 83.30 | 81.35 | **1.02** |
| | $Camp_{Kawlin}$ | -196.41 | 26.39 | **-7.44** |
| | $Camp_{Mandalay\_Magway}$ | -161.91 | 67.90 | **-2.38** |
| | $Camp_{Naypyidaw\_Bago}$ | -243.93 | 50.30 | **-4.85** |
| | $Camp_{Sagaing}$ | -273.63 | 43.78 | **-6.25** |
| | $Camp_{Shan}$ | 27.88 | 50.41 | **0.55** |
| | $Camp_{West\ Katha}$ | -51.76 | 31.58 | **-1.64** |
| | $Origin_{captive}$ : Birth $season_{hot}$ | 33.12 | 22.79 | **1.45** |
| | $Origin_{captive}$ : Birth $season_{monsoon}$ | 96.16 | 23.53 | **4.09** |

**Table A3**. Model selection testing the seasonal effect on FCMs in analyses without the calves and juveniles using mixed models. We performed a model selection. *Base* model contains fixed (*F()*) and random (*R()*) confounding variables. FCMs are proxies of the stress level. We used gaussian distributions with a log-link. We provide the number of parameters (K), the corrected AIC (AICc), the difference in AIC between the focal and the best fitting models (ΔAICc), the AIC weight (AICcWt). The best fitting model is highlighted in grey.

| **Trait** | | **K** | **AICc** | **ΔAICc** | **AICcWt** |
|---|---|---|---|---|---|
| **FCMs** | *Base: FCM ~ F(sex)+F(age)+F(camp)+F(origin)+F(birth season)+R(1\|year/ID)* | | | | |
| (1851 measures; | Base + Season | 14 | 7301.80 | 0.00 | 0.93 |
| 261 individuals) | Base | 12 | 7307.04 | 5.23 | 0.07 |

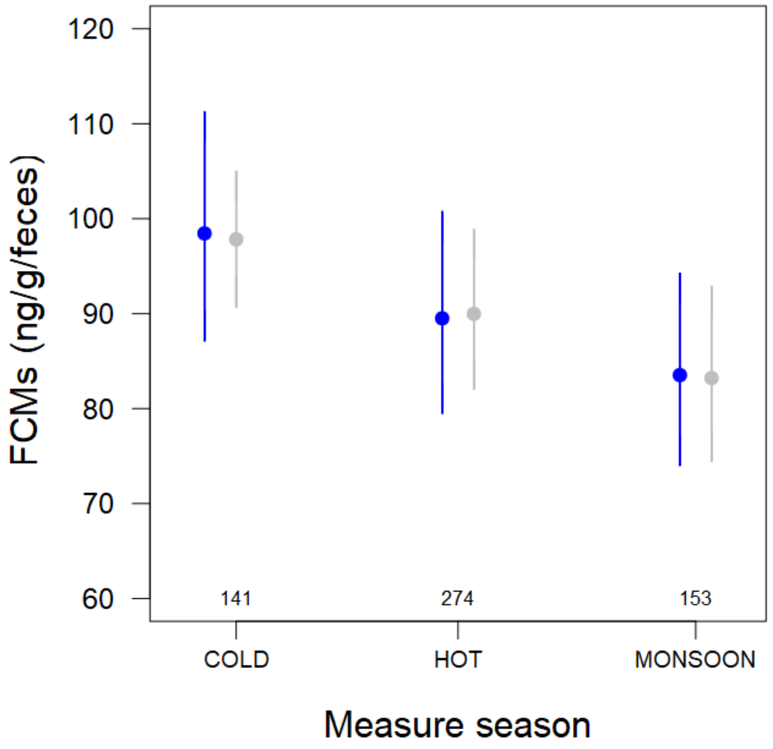


**Figure A1**. We showed seasonal variation in FCMs level in analyses without the calves and juveniles. The figure presents predicted values (blue dots) from the linear mixed models with SE bars and observed means (grey dots) with [95]CI error bars, and the number of repeated measurements for each season.
